# Unveiling changes in the landscape of patient populations in cancer early drug development

**DOI:** 10.18632/oncotarget.13258

**Published:** 2016-11-09

**Authors:** Cinta Hierro, Analía Azaro, Guillem Argilés, Elena Elez, Patricia Gómez, Joan Carles, Jordi Rodon

**Affiliations:** ^1^ Department of Medical Oncology, Vall d'Hebron University Hospital, Vall d'Hebron Institute of Oncology, Barcelona, Spain

**Keywords:** molecularly targeted agents, patient populations, window-of-opportunity, healthy volunteer, phase 0

## Abstract

The introduction of new Molecularly Targeted Agents (MTA) has changed the landscape in Early Drug Development (EDD) over the last two decades, leading to an improvement in clinical trial design. Previous Phase 1 (Ph1) studies with cytotoxics focused on safety objectives, only recruiting heavily pre-treated cancer patients, have been left behind. In this review, we will illustrate the slow although unstoppable change that has increasingly been observed in those populations candidate to participate in EDD trials with the advent of MTA. As more evidence regarding oncogene addiction becomes available, molecular-biomarker driven selection has been implemented among Molecularly-Selected Population (MSP) studies. New Window-Of-Opportunity (WOO) and Phase 0 (Ph0) studies have been developed in order to assess whether a MTA produces the hypothetical proposed biological effect. The rising need of getting early pharmacokinetics and pharmacodynamics data has led to the conduction of Healthy Volunteer (HV) studies, in part favoured for the particular and different toxicity profile of these MTA. However, several challenges will need to be addressed in order to boost the implementation of these new clinical trial designs in the forthcoming years. Among the problems to overcome, we would highlight a better coordination effort between centers for ensuring adequate patient accrual among small patient populations and a deepening into the ethics implied in enrolling patients in studies with no therapeutic intent. However, these tribulations will be certainly compensated by the possibility of opening a new horizon of treatment for diseases with dismal prognosis.

## INTRODUCTION

Over the past two decades, with the advent of new Molecularly Targeted Agents (MTA), cancer research has shifted from standard chemotherapies to the selective inhibition of signaling pathways. However, while some of these drugs have offered a significant breakthrough, new insights into the tumor biology and drug development are still required to further delineate rational therapeutic strategies [1]. Phase 1 (Ph1) trials with cytotoxics are mainly focused on safety. Traditionally, first-in-human (FIH) studies were designed with a 3+3 dose escalation based on acute toxicities. Considering risks and limited efficacy, only heavily pre-treated cancer patients with no standard effective therapies were considered candidates. This dose-toxicity model, though, has shown to be not so effective for testing MTA.

The introduction of MTA has changed the landscape in Early Drug Development (EDD) and subsequently, the clinical trial design. Molecular biomarker-driven selection of patients has progressively been incorporated, as more evidence regarding oncogene addiction is available [2]. New dose escalation strategies are followed now, using dose recommendation methods that help in decision making by providing the probability of toxicities or by incorporating chronic toxicities [3, 4]. Also, there has been a better integration of human pharmacokinetics (PK) and pharmacodynamics (PD). In fact, several new trial designs can be now successfully implemented in the EDD process further to the classical 3+3 design, such as the accelerated titration design (ATD) and continual reassessment method (CRM) [4]. This new designs take advantage of incorporating supplementary endpoints that help to further characterize the MTA in terms of efficacy (mechanism of action -MoA-/PD biomarkers) on top of the traditional toxicity endpoints, and also allow a fine-tune assessment of the safety profile by evaluating the late-onset toxicities and not only the acute ones. Finally, there is increasing evidence that some of these MTA present a class-specific toxicity, related to their “on-target” effect (e.g.: fibroblast growth factor receptor inhibitors and hyperphosphatemia)[5]. As target inhibition can cause toxicity leading to treatment interruptions, the consideration of new optimal schedules while maintaining the target inhibition-related anti-tumor efficacy has become a critical key point [5].

Within this context, we are progressively witnessing an unprecedented shift towards a completely different population profile in EDD. To clarify in which selected biological context the drug works, some trials include now Molecularly Selected Patient (MSP) populations [2]. Despite the efforts, there are still scarce predictive biomarkers, meaning that a rising need to determine upfront whether a MTA produces the hypothetical biological effect exists. New Window-Of-Opportunity (WOO) studies [6] and Phase 0 studies (Ph0) [7, 8] have been developed in order to fill in the gap. MTA have revolutionized previous toxicity concepts, as may not have single direct dose-response relationship, with higher doses lacking of further benefit after a biological effective dose has been reached [9]. Longstanding mild toxicity is commonly seen instead of acute intense toxicities, with neither teratogenicity nor carcinogenic effects. Considering their different toxicity profile [10], MTA have favoured the conduction of Healthy Volunteer (HV) studies [11, 12], aiming for PK and PD endpoints.

Based on these premises, we will illustrate the change in the population of patients enrolled in early clinical trials testing MTA.

## MOLECULARLY- SELECTED POPULATION STUDIES

Some MTA have been already approved despite the fact of not having identified a predictive biomarker to associate their clinical efficacy. Multi-tyrosine kinase inhibitors, such as sorafenib in hepatocarcinoma [13], and sunitinib in renal cancer [14] would fall into this category. But for the majority of these MTA, it has been key in their development to tailor them with molecular aberrations that harbor different tumor types. Therefore, pre-identification of a driver alteration has become mandatory to prescribe these MTA. Overexpression or amplification of HER2 in breast or gastric carcinomas [15], EGFR mutations in lung carcinoma [16], and BRAF mutations in melanoma [17], would be the best examples of tailored tumor-type treatments. One must bear in mind that whilst some of the MTA target molecular alterations that have not been proven to be drivers for any specific disease (e.g. PI3KCA mutations), other MTA target clear defined oncogenic aberrations (e.g. EGFR mutations in non-small cell lung cancer –NSCLC-). The molecular selection of patients will be key in the second case for warranting the success of a MTA in that subset of patients, but less obvious and not mandatory in the first case.

Noteworthy, some MTA have been recently approved after the initial studies showed impressive response rates in subsets of MSP. Interestingly, some of these first MTA were not initially developed for a pre-specified MSP, but as the Ph1 was being conducted, early signs of response were noticed in genomically-altered subgroups of patients. The later enrichment confirmed the oncogenic addiction for a specific aberration, which lead to the MTA approval only for those subsets of patients. That is the case of crizotinib for NSCLC with ALK rearrangement [18].

Ph1 clinical trials have emerged as the most suitable arena for testing the hypothetical relationship between a predictive biomarker and the efficacy of a given drug, driving knowledge in the right direction. The idea for doing this very early in the development of a drug is to test the efficacy in the best-case scenario and for assisting in *go-no go* decisions [19]. By enriching early Ph1 trials with patients harboring specific molecular alterations, it could be demonstrated the proof-of-concept hypothesis for the MTA mechanism and encourage its further development [20]. But quite frequently, these markers are not well validated, and to date, they are still used as enrichment biomarkers. Nevertheless, the discovery of robust predictive biomarkers in subsets of patients parallel to the development of MTA, could provide a personalized therapeutic option for cancer patients lacking of standard options. As highlighted before, that was the case with crizotinib, the first-in-class ALK inhibitor, that demonstrated fast and durable responses in metastatic ALK positive NSCLC patients in early Ph1 [18, 21] and Ph2 [22] trials. These results lead to the accelerated crizotinib-FDA approval after a three-year development programme, and opened the door to the pharmaceutical companies to apply for a Fast Track designation from the regulatory agencies. Recently, the approval of ceritinib for NSCLC with ALK rearrangement after progression to crizotinib, solely based on the results of the Ph1, has supposed another milestone in the history of EDD in Oncology [23]. This clearly shows that well conducted Ph1 trials could accelerate the drug development, especially where a drug could fill a void for a poor prognosis orphan disease. This really depends on how well designed are these early clinical trials, specially in terms of wise collection of the generated data in order to prove the MoA of the new MTA and to allow the analysis of statistically meaningful preliminary data. Table 1 depicts some of the most relevant approved MTA. These MTA have been selected in view of the impact that their development represented for the clinical management of these tumor subtypes.

**Table 1:** Some relevant approved MTA developed with and without a pre-specified Molecularly-Selected Population (MSP)

Family of MTA	MTA	Target	Genetic alteration as inclusion criteria in Ph1	Early signs of efficacy in Ph1	Ref	Metastatic tumor type	FDA approval study
**Anti-HER2**	**lapatinib**	EGFR1 HER2	Yes, EGFR1/HER2over	No CR 4 PR (BC HER2+ TTZresist)	[60]	BC HER2+	Ph3 [61]
	**pertuzumab**	HER2	No	No CR 2 PR (1 OC, 1 PC)	[62]	BC HER2+	Ph3 [63]
**Multi-tyrosine kinase inhibitors**	**imatinib**	BCR-ABL c-KIT	No. Study only in soft-tissue sarcomas. All enrolled GIST c-KITover	No CR 16 PR (all GISTs)	[64]	GIST	Ph3 [65–67]
**EGFR** **inhibitors**	**erlotinib**	EGFR	No	1 CR (RCC) 1 PR (CRC) 6 SD	[68]	AdeNSCLC EGFRmut	Ph3 [69]
	**gefitinib**	EGFR	No	No CR No PR 7 SD	[16]	AdeNSCLC EGFRmut	Ph3 [70]
**PARP inhibitors**	**olaparib**	PARP	No, expansion enriched with BRCAmut	20 CR/PR (OC) 3 SD	[71]	OC BRCAmut	Ph2 [72]
**BRAF inhibitors**	**vemurafenib**	BRAF	No, expansion enriched with M BRAFmut	3 CR 34 PR (M)	[73]	M V600E BRAFmut	Ph3 [74]
	**dabrafenib**	BRAF	No, later on only BRAFmut tumors	No CR 20 PR (18 M, 1 NSCLC, 1 CRC)	[17]		Ph3 [75]
**ALK inhibitors**	**crizotinib**	ALK MET	No, expansion enriched with NSCLC ALKtransl	3 CR 84 PR (NSCLC ALKtransl)	[18]	AdeNSCLC ALKtransl	Ph2 [22]
	**ceritinib**	ALK GF-1	Yes, ALKtransl tumors	1 CR NSCLC 67 PR (65 NSCLC, 1 ALCL, 1 myofibroblastic tumor)	[23]		Ph1 [23]

*Abbreviations*: MTA (Molecularly Targeted Agents); HER2 (Human Epidermal growth factor Receptor 2); EGFR (Epidermal Growth Factor Receptor); ALK (Anaplastic Lymphoma Kinase); PARP (Poly [ADP-ribose] polymerase); BRCA (BReast CAncer gene); over (overexpression); ampl (amplification); mut (mutation); transl (translocation); BC (Breast Cancer); HR+ (Hormone Receptor positive); TTZresist (TrasTuZumab resistant); OC (Ovarian Cancer); PC (Pancreatic Carcinoma); GIST (GastroIntestinal Stromal Tumors); RCC (Renal Clear Cell carcinomas); CRC (ColoRectal Carcinomas); M (Melanomas); NSCLC (Non Small Cell Lung Cancer); Ade (Adenocarcinoma); ALCL (Anaplastic Large-Cell Lymphoma); CR (Complete Response); PR (Partial Response); SD (Stable Disease); Ph (Phase).

In parallel, we are witnessing the development of new molecular diagnostic techniques alongside to the discovery of new aberrations. This fact has led to a gradually narrowing of the pre-selection criteria for candidate patients. Sometimes, a specific molecular alteration is not a requirement for participating in a study, but throughout the trial, an increasing number of patients with that aberration show response, and this biomarker is later on required for inclusion. In other cases, Ph1 trials do pre-select patients according to a specific molecular alteration from the early beginning. In both situations, the tactics allows the study to enrich the population, and might help us to early identifying the subgroup of patients who will benefit the most from the MTA. Some of the MTA that are contemporary being developed under strict molecular pre-selection criteria are shown in Table 2.

**Table 2:** Some ongoing early clinical trials testing MTA in Molecularly-Selected Populations (MSP)

Family of MTA	MTA	Target	Genetic alteration as inclusion criteria in Ph1	Early signs of efficacy in Ph1	Ref	Ongoing studies	ClinicalTrials.gov identifier
**FGFR inhibitors**	**AZD4547**	FGFR1/2/3	Yes, since escalation, FGFR1/2ampl	No CR 2 PR (SqNSCLC FGFR1ampl, GC FGFR2ampl) SD (BC, GOJ/GC SqNSCL, UC FGFRampl)	[76–78]	Ph1 Ph2 NSCLC Ph2 GC/GOJ Ph2 BC	NCT00979134 NCT01824901 NCT01457846 NCT01202591
	**BGJ398**	FGFR1/2/3	Yes, since escalation, any FGFRalt	No CR 6 PR (4 UC FGFR3mut, 2 SqNSCLC FGFR1ampl) SD (BC FGFR1ampl, ABTC FGFR2transl)	[79]	Ph1 Ph2 ABTC	NCT01004224 NCT02160041
	**JNJ-42756493** **(erdafitinib)**	FGFR1/2/3/4	Yes, since escalation, any FGFRalt	1 CR (UC FGFR2trunc) 1 PR (UC FGFR3transl) SD (lung, CS, BC FGFR1ampl)	[80]	Ph1 Ph2 UC	NCT01703481 NCT02365597
**MET inhibitors**	**INC280** **(capmatinib)**	MET	Yes, since escalation, METalt	No CR No PR SD (CRC, HCC, lung)	[81]	Ph1 Ph2 NSCLC	NCT01324479 NCT01911507
	**SAR125844**	MET	Yes, since escalation, METalt	No CR 1 PR (lung cMETampl) SD (not specified)	[82]	Ph1 Ph2 NSCLC	NCT01391533 NCT02435121
**EGFR inhibitors**	**PF-00299804** **(dacomitinib)**	Pan-HER	Yes, since expansion, EGFRalt	No CR 4 PR (AdeNSCLC) SD (AdeNSCLC, others)	[83]	Ph1 Ph2 OEC Ph3 NSCLC	NCT00225121 NCT01608021 NCT01000025
**Multi-tyrosine kinase inhibitors**	**E3810** **(lucitanib)**	VEGFR1/2/3 PDGFRa/b FGFR1/2/3	Yes, since expansion, cohort FGFR1/11q ampl	3 CR (2 MTC, 1 RCC) PR (NSCLC and BC FGFR1/11q ampl) SD (NSCLC and BC FGFR1/11q ampl, others)	[84]	Ph1 Ph2 BC HR+ Ph2 NSCLC	NCT01283945 NCT02053636 NCT02109016
**PIK3CA/** **mTOR pathway inhibitors**	**BYL719** **(alpelisib)**	PIK3CAa	Yes, since escalation, PIK3CAalt	No CR 15 PR (2 BC HR+ PIK3CAalt)	[85]	Ph1 Ph2 BC HR+	NCT01219699 NCT02058381
	**BKM120** **(buparlisib)**	Pan-Class I PIK3CA	Yes, since expansion, cohort PIK3CA/PTENalt	No CR 4 PR (1 confirmed TBNC PI3KCAmut, 1 PGC, BC HR+,EH)	[86]	Ph1 Ph2 TBNC	NCT01068483 NCT01629615
**MAPK pathway inhibitors**	**LGX818** **(encorafenib)**	BRAF	Yes, since escalation, BRAF V600mut	No CR 13 PR (M BRAF V600mut), 12 SD (CRC BRAF V600mut)	[87, 88]	Ph1	NCT01436656
	**MEK162** **(binimetinib)**	MEK1/2	Yes, since expansion, KRAS and BRAFmut	No CR 1 PR (ABTC NRASmut), 9 SD	[89]	Ph1	NCT00959127
**PARP inhibitors**	**olaparib**	PARP	No, expansion enriched BRCAmut	1 CR (BC) 21 PR (15 OC, 6 BC)	[90]	Ph1 BC/OC Ph2 TBNC Ph3 OC	NCT01445419 NCT02681562 NCT02446600
	**talazoparib**	PARP	Yes, since expansion, BRCAmut	No CR 13 PR (11 OC/peritoneal, 2 BC BRCAmut)	[91]	Ph1 Ph2 BC	NCT01286987 NCT02034916
**ROS/ALK inhibitors**	**X-396**	ALK	Yes, since escalation, ALKalt	No CR 5 PR (NSCLC ALKtransl) 5 SD (NSCLC ALKtransl)	[92]	Ph1	NCT01625234
	**RXDX-101** **(entrectinib)**	Pan-TrKA/B/C ROS1 ALK	Yes, since escalation, TrKA/B/C, ROS1 and ALKalt	No CR 4 PR (1 CRC TrKA+, 1 NSCLC ROS1+ and NSCLC ALK+, 1 NB ALK+) 2 SD (NSCLC ALK+, PC ROS1+)	[93]	Ph1	NCT02097810

*Abbreviations*: MTA (Molecularly Targeted Agents); HER 2 (Human Epidermal growth factor Receptor) 2; EGFR (Epidermal Growth Factor Receptor); ALK (Anaplastic Lymphoma Kinase); FGFR (Fibroblast Growth Factor Receptor); ampl (amplification); mut (mutation); trunc (truncation); transl (translocation); alt (alteration); HCC (HepatoCarcinoma); NSCLC (Non Small Cell Lung Cancer); Ade (Adenocarcinoma); Sq (Squamous); ALCL (Anaplastic Large-Cell Lymphoma); BC (Breast Cancer); HR+ (Hormone Receptor positive); CS (ChondroSarcoma); OEC (OEsophageal Carcinoma); GOJ (GastroesOphageal Junction carcinoma); GC (Gastric Cancer); UC (Urothelial Carcinoma); MTC (Medullary Thyroid Carcinoma); RCC (Renal Clear Cell carcinoma); M (Melanoma); CRC (ColoRectal Cancer); ABTC (Advanced Biliary Tract Carcinoma); OC (Ovarian Carcinoma); TBNC (Triple Breast Negative Cancer); PGC (Parotid Gland Carcinoma); EH (Epithelioid Hemangiothelioma); NB (NeuroBlastoma); PC (Pancreatic Carcinoma); CR (Complete Response); PR (Partial Response); SD (Stable Disease); VEGFR (Vascular Endothelial Growth Factor Receptor); PDGFR (Platelet Derived Growth Factor Receptor); TrK (Tropomyosin receptor Kinase); PARP (Poly [ADP-ribose] polymerase); BRCA (BReast CAncer gene); Ph (Ph).

Recently, the results of a meta-analysis presented by Schwaederle MC. et al [24] showed improved outcomes in patients treated in Ph1 trials using a biomarker-selection strategy. The analysis of over 13.203 patients demonstrated that a more personalized approach had a statistically improved RR (30.6% versus 4.9%, p<0.0001) as well as a better PFS (5.7 months versus 2.9 months, p=0.002) compared to the non-personalized option. These data reflect the importance of delineating a better biomarker-selection of patient candidates to participate in early clinical trials developing MTA, as the majority of Ph1 testing targeted agents in non-selected patients presented trivial responses.

Of note, analyzing a tumor sample can easily change the selected population. Firstly, where a fresh tumor sample is required, patients deemed not biopsiable might have less chances of trial inclusion. Also, patients are at risk of deteriorating while waiting for a complex biomarker assessment (central review), biasing the trial towards healthier patients, and notice that inadequate or too strict molecular pre-screening criteria may considerably slow down the recruitment. Secondly, patients whose tumor bears the pre-specified alteration may be recruited even if standard therapies have not been exhausted yet, based on increased expectations for response. It is worth noticing that not all patients that are tested positive for the selection biomarker would be finally considered eligible for a trial, therefore molecular selection can sometimes generate false hope for patients. Finally, patients may be excluded based on an insufficiently validated biomarker.

In fact, the identification of suitable biomarkers has become one of the main priorities in parallel with the development of new MTA, although it has proven to be challenging [25]. Predictive biomarkers are patient and/or disease characteristics that can be objectively measured, indicating subgroup of patients who are most likely to benefit from a therapy [26, 27]. Whenever developing a biomarker, researchers have to keep in mind that a feasible assay should be validated according to a “fit-for-purpose” approach, in order to ensure accuracy and reproducibility of the detection assay [28]. Nevertheless, differences in sample acquisition and processing, assay implementation and analysis across different centers, can be counted as certain of the hurdles to overcome. In addition, knowing the intrinsic tumor heterogeneity [29], it should be considered that a negative biomarker sample does not specifically mean that the patient cannot yet benefit from the study drug.

We are facing an era where there will be increasing evidences that several tumors could be categorized by specific molecular changes that drive their proliferation. The hypothesis that blocking an activated oncogenic pathway will lead to tumor control warrants further efforts to determine biomarkers for facilitating this patient population identification. In our opinion, a better delineation of genomic pre-screening strategies seems to be the right direction for identifying those patients who may achieve greater benefit from an MTA, and could avoid unnecessary toxicities to patients who are less likely to benefit from this drugs, minimizing the risks and developmental costs of highly expensive targeted therapies. However, one should be aware of the level of validation of the selection biomarker and its companion diagnostic assay, before switching the participating patient population towards less pre-treated patients.

## WINDOW- OF- OPPORTUNITY STUDIES

The development of MTA and the need of testing their biological effect have highlighted the convenience of implementing new trial designs, considering that the MTA activity may be shadowed in advanced oncology patients, because of previous cytotoxic resistances, residual toxicities or high tumor burden conferring high intra-tumor heterogeneity. WOO studies have been considered one of the best scenarios for testing MTA and biomarker development, as patients receive the MTA for a short period of time -concept of window- just before performing the standard treatment. Aiming to evaluate the key target modulation accordingly to the drug exposure levels, the objective is to deepen into the proof-of-mechanism anti-tumor activity of the MTA in a cancer stage that is not altered by previous therapies. The main goals are to confirm the biological effect of the MTA and its PK properties, together with validating possible biomarkers that could predict subgroups of patients most likely to benefit. However, no clinical benefit is pursued for the treated patients [30].

WOO trials have been developed in the neo-adjuvant setting [31] but differ from traditional neo-adjuvant trials [32] in the limited treatment duration, the non-therapeutic intent, and the focus on biomarkers of the former MTA in comparison to the use of pathological complete response (pCR) of the latters [33]. Whilst traditional neo-adjuvant trials focus on evaluating the anti-tumor activity of a certain drug by assessing the pCR rate, WOO studies aim to further characterize the biological activity of the new experimental therapy with PK/PD objectives as principal end-points.

WOO studies allow obtaining an unperturbed tissue specimen, appropriate to validate pathway inhibition by the MTA in the same tumor sample and to assess potential compensating feedback loops to better understand cross-talked resistance pathways. WOO studies may represent proof-of-biological efficacy/mechanism studies which serve as an early step before further phase 2/3 proof-of-clinical efficacy studies [34]. Basically, WOO neo-adjuvant studies among women with early large primary breast cancers have been performed with this design [6], as depicted in Table 3. As these WOO studies have changed the concept that curable patients may be treated with an experimental drug for interrogating biological questions, breast cancer patients have been considered one of the most feasible settings considering that radical surgery of the primary tumor is often indicated as first therapeutic option.

**Table 3:** Some ongoing Window-Of-Opportunity (WOO) studies testing MTA in the neo-adjuvant setting

Family of MTA	MTA	Mechanism of action	Endpoints of the study	Subject dose	Status (histology)	ClinicalTrials.gov Identifier or Reference
**AKT inhibitors**	MK-2206	AKT inhibitor	To assess pAKT modulation in tumor tissue.	MK-2206 dose day -9/day -2	Ph2 (early BC)	[94]
**IGF-IR inhibitors**	CP751871 (figitumumab)	Monoclonal antibody for IGF-IR	To assess tumor total choline changes determined by MRS.	CP751871 dose day 1/22	Ph1 withdrawn (early BC)	NCT00635245
**IGF-IR**	CP751871 (figitumumab)	Monoclonal antibody for IGF-IR	To assess biological RR (proportion of patients with inhibition of IGF-IR expression by IHC).	CP751871 20 mg/kg every 3w x3 cycles	Ph2 (early PrC)	[95]
**mTOR inhibitors**	MK-0646 (dalotuzumab) with or without MK-8669 (ridaforolimus)	Monoclonal antibody for IGF-IR mTOR inhibitor	To demonstrate inhibition of GFS in patients with high proliferation index.	A) MK-8669 OD x5 days/week and MK-0646 once weekly. B) MK-8669 OD x5 days/week. C) MK-0646 once weekly	Ph1 (early BC)	NCT01220570
**Oral anti-diabetics**	metformin	Metabolic signalling pathway inhibition (cAMP, protein kinase A)	To evaluate changes in proliferation marker (Ki67) in tissues.	Daily dose at bedtime for 2 weeks, prior planned surgery	Ph0 (early BC or DCIS)	NCT01980823

*Abbreviations*: MTA (Molecularly Targeted Agents); pAKT (phospho-AKT); IGF-IR (Insulin-like Growth Factor 1 Receptor); MRS (Magnetic Resonance Spectroscopy); Ph (Phase); BC (Breast Cancer); RR (Response Rate); IHC (ImmunoHistoChemistry); PrC (Prostate Cancer); GFS (Growth Factor Signature); OD (Once Daily); DCIS (Ductal breast Carcinoma *In Situ*).

Interestingly, many WOO studies are usually performed just after the exploration of a MTA in FIH studies. This observation reflects two issues: firstly, that biological activity might be a key aspect in EDD, assisting in *go-no go* decisions very early in the drug development process. Secondly, curable patients are exposed to experimental MTA that have been previously explored in small cohorts of patients. While this last observation may be due to the relatively favorable profile of these MTA, it also reflects a change in the paradigm of the type of patients enrolled in clinical trials with experimental drugs.

But despite all the advantages that represent the WOO studies, why have they not been routinely implemented for drug development so far? WOO studies have been widely debated, considering the ethical implications involved in delaying upfront standard approved therapies. However, in our opinion, WOO trials emerge as a very useful tool for facilitating the accelerated development program of a certain drug, shortening the laborious process of evaluating a new MTA. As long as safety monitoring and careful candidate pre-selection are performed, WOO studies may optimize and increase the chances of success of a specific MTA from the early beginning, as the failure to achieve the predefined objectives in the study may be a sign for not continuing with its development. Also, it has to be considered that patients included in WOO trials may truly reflect the real intention-to-treat population compared to the heavily pre-treated Ph1 patients. Nevertheless, intense efforts should be done in order to ensure that patients understand the risk/benefit ratio of participating in these clinical trials, to warrant precocious detection of disease progression and to avoid enrolling unfit patients [35]. Maybe because of this risk/benefit ratio, most of the WOO trials testing MTA in the neo-adjuvant setting have included well-known drugs. In reality, as depicted in Table 3, very few of them include experimental drugs in early stages of their development, with most of the WOO studies published in the literature examining drugs that are already transitioning to the late development process.

Despite their appealing potential, moving an experimental therapy with a limited long-term toxicity profile experience to the neo-adjuvant setting, with curable untreated patients, may be carefully considered. That is the reason why WOO studies should only be considered and acceptable in populations with a high risk of relapse and a significant unmet medical need (e.g. patients with high grade, hormone receptor positive and HER2-negative breast cancers). It should be kept in mind that not all drugs are suitable for being tested in a WOO trial, as only some drugs with a plausible biological MoA thought to be involved in cancer modulation, with a well-described toxicity profile and displaying particular PK/PD characteristics, can be considered good candidates for being explored in this setting. As example, the oral anti-diabetic drug, metformin, is currently moving towards a new classification as a potential cancer metabolism-targeted drug, due to the underlying link between obesity and hyper-insulinemia and breast cancer [36]. However, the clinical translation of metformin as a new anti-cancer therapy has been limited by the lack of PD biomarkers (e.g. Ki67 % or proliferative index) that could translate into an early read out of its biological activity in cancer patients so far. On top of that, as most of the preclinical studies do not have an appropriate PK design, some of the limited results obtained in *in vivo* and *in vitro* experiments may be related to the suboptimal doses tested of metformin [37]. One of the strengths of metformin is that its short and long-term toxicity effects are already well-described, and the drug is deemed safe and widely implemented in the daily care of millions of diabetic patients. Testing metformin in a WOO clinical trial design [6] could provide more insights into the clinical PK/PD of the drug in an optimal model that mimics the real patient scenario. The WOO strategy will help to better illustrate its PD mechanism of action (whether the administration of metformin reduces the proliferation of tumoral cells by assessing the Ki67 proliferative index in surgical specimens) and to optimize a relevant and achievable drug concentration in plasma by obtaining detailed PK data. Therefore, the PD/PK data will help in delineating future large scale randomized clinical trials, maximizing the chance of success of metformin as a new anti-cancer strategy.

## PHASE 0 STUDIES

By definition, Ph0 trials are clinical studies conducted in EDD before the traditional Ph1 studies, to provide human PK and PD data, benefiting EDD by allowing to clinically testing a proposed MoA. They are FIH trials with neither therapeutic nor diagnostic intent, with a low number of patients included and limited drug exposure. Part of the rationale for conducting them is that the new agent promises significant biological effect in a substantial percentage of participants, even at doses below the expected ones that could lead to toxicity [38]. However, several limitations of the Ph0 have made highly controversial their implementation, such as their intense resource use and complexity, the request of serial tumor biopsies, the requirement of analytical methods that are not routinely available, and specially, the lack of therapeutic purpose for the treated patients [39].

The Ph0 concept implies a change in role of studies in Oncology. In Ph0 studies, candidates assume the role of “sick volunteers” given the absence of any direct efficacy for the participant, closer to the HV concept –see next section- [40]. The main motivator may be altruism for the “benefit to others”. Hence, Ph0 trials are both ethically challenged and challenging, since the patient has to understand that clinical benefit is not pursued, and that even if the risks seem low, there is a high degree of uncertainty regarding potential toxicities [41]. Under these circumstances, only patients with advanced incurable malignancies should be recruited after failure of standard therapy or in case that their indolent diseases do not require immediate treatment. Furthermore, not all MTA can be explored within a Ph0 context. Ideally, one must have a minimum of toxicology package and certain knowledge of the PK properties of the drug evaluated. Previous extensive preclinical experiments are required to establish a safe starting dose and schedule, in order to help in predicting the plasmatic levels of the MTA (PD data) that are required for target modulation (PK data) without reaching a therapeutic level [42].

Despite the fact that some experts in EDD consider that such trials will become a routine of the field in the future [42], we could only find few examples of contemporary trials with this design, probably because of the previously mentioned challenges and that similar information can be achieved with the regular Ph1 trial designs. It is worth noticing that many of them were published as Ph0 trials, although they do not fulfill the criteria for a Ph0 design, as shown in Table 4. Most of these studies include drugs with a significant clinical experience, which should be called “biomarker-driven” studies. Among the properly Ph0 trials detected, new imaging compounds have been tested, such as the 11C-labeled topoisomerase I/II inhibitor N-[2-(dimethylamino)ethyl]acridine-4-carboxamide (DACA) [43]. Ph0 trials could be also focused on determining pharmacologically relevant doses of a certain drug, such as imatinib [44], or they could assess the MoA/PD markers of new compounds, like the ABT-888 [7] or the STAT3 decoy oligonucleotide [8].

**Table 4:** Examples of Phase 0 (Ph0) studies testing MTA in Oncology

Family of MTA	MTA	Mechanism of action	Endpoints of the study	Subject dose	Fulfils Ph0 criteria	Reference
**Cytotoxic agent radiolabelled with positron emitting radioisotopes**	**carbon-11 radiolabelled** **N-[2-(dimethylamino)** **ethyl]acridine-4-carboxamide (DACA)**	11C-labelled topoisomerase I/II inhibitor	To evaluate plasma PD effects of drugs using data obtained during PET studies with radiolabelled anti-cancer agents.	DACA at 1/1000th of Ph1, as part of Ph0 micro-dosing study	Yes	[43]
**Multi-tyrosine kinase inhibitor**	**imatinib**	BCR-ABL and c-KIT inhibitor	To investigate the potential use of MS for studying pharmacology aspects of imatinib.	Imatinib 400 mg/d plus 13.6 kBq of 14C-imatinib	Yes	[44]
**PARP inhibitor**	**ABT-888** **(veliparib)**	Poly (ADP-ribose) polymerase inhibitor	Proof-of-mechanism of action. To evaluate PARP levels after dosing (PD effect).	Starting dose 1/50th of NOAEL of sensitive specie	Yes	[7]
**Transcription factor inhibitor**	**STAT3 decoy oligonucleotide**	STAT3 transcription factor gene inhibition	Proof-of-mechanism of action. To demonstrate inhibition of STAT3 target genes (Bcl, Cyclin D).	Single intra-tumoral injection of several doses: 250 mg/250uL vs 500 mg/500 uL vs 1000 mg/1000 uL	Yes	[8]

*Abbreviations*: MTA (Molecularly Targeted Agents); PD (PharmacoDynamic); MS (Mass Spectrometry); PARP (Poly [ADP-ribose] polymerase).

Further efforts to improve their design limitations seem mandatory, as they may be a promising field to get data from a new compound in early phases of its development.

## HEALTHY VOLUNTEER STUDIES

Outside the Oncology field, it is common that the introduction of a new drug in patients is tested firstly in HV, limiting human exposure. Designing the appropriate FIH dose requires close collaboration among the toxicologists and the preclinical scientists, to use modeling data from animal experiments to determine a safe starting dose and a dosing interval [45]. The definition of HV per se is challenging. The widely accepted definition comprises those healthy, adult volunteers, in well-defined and controlled conditions. However, this definition underlies several margins of discretion regarding the wellness of a patient, and a critical judgment is mandatory when enrolling a candidate. Several ethical concerns arouse in 2006 with The TeGenero incident [46], where TGN1412 caused life-threatening toxicities and fostered the implementation of strict guidelines for EDD. Subsequently, specific rules were developed to guide HV studies [47]. These HV studies should be conducted in units with sufficient expertise, and their usual characteristics include: 1) A single subject receives the first dose within a justified period of observation before the next subject receives another dose, 2) double-blind design is preferred to avoid risks of bias, 3) subjects are randomly assigned to receive either the active drug or the placebo/control drug, and 4) different designs are used (parallel, crossover and sequential groups) based on the objectives of the study.

In Oncology, though, most anticancer agents have been tested only in advanced cancer patients assuming their potential severe side effects, mutagenicity and carcinogenesis, accepting higher degrees of toxicity considering that these secondary effects would be less threatening than the disease itself [48]. Interestingly, new MTA have started to be explored in HV studies recently. This is probably based on their relatively broaden safety profile and because these HV are more able to withstand any unexpected toxicity from these new molecular entities [49]. However, careful consideration must be taken in view of the novelty of their MoA. We may not fully know the exact MoA for a specific MTA by the time we initiate early studies, therefore special attention is required in view of possible interactions between biological cascades. The risk with MTA could emerge not only from their chemical structure but also from their biologic or intended pharmacological mechanisms [50]. As example, some MTA have revealed delayed severe side effects (e.g. pneumonitis with mTOR inhibitors [51, 52]) and many combinations of MTA have proven to be challenging in terms of overlapping toxicities [53, 54].

The types of studies done in HV in Oncology include:

1. Drug-drug and food-drug interaction studies, conducted to investigate what concomitant medications or food/drink products can interfere with the pharmacokinetic characteristics of a tested MTA [11, 12].
2. Cardiovascular safety studies are required if the new MTA shows potential cardiovascular toxicity in preclinical animal studies, especially changes in the repolarization based on drug-induced inhibition of potassium channels [55, 56]. If feasible, these studies are strongly recommended to perform in HV, with far less inter-subject variability and without interference by any other adjacent comorbidity or concomitant medication.
3. Bioavailability and bioequivalence studies are developed in order to analyze the effect of changes in the physiochemical properties of a drug, like comparing different formulations and dosage forms, or assessing bioequivalence between several medicinal products containing the same active substance [57]. As MTA are complex molecules, the new ones may not be identical although potentially “biosimilar” to the marketed drugs [58].
4. Finally, classical dose-escalation FIH studies in HV pretend to assess safety and tolerability of a new MTA, considering escalating single or multiple doses [59]. These types of studies do not differ from Ph1 studies done in other disciplines, such as in Endocrinology.

Some examples of MTA tested in each subtype of HV studies are represented in Table 5. Remarkably, HV studies imply a substantial change in patient populations in EDD, since HV represent the opposite side of the spectrum compared with the classical refractory cancer patients.

**Table 5:** Examples of Healthy Volunteer (HV) studies testing MTA

Type of HV study	MTA	Mechanism of action	Endpoints of the study	Healthy subject dose	Status	ClinicalTrials.gov identifier
**Drug-drug**	**PF-00299804** **(dacomitinib)**	Pan-HER inhibitor	Pharmacokinetic. To asses interactions between paroxetine and dacomitinib.	Single doses 45 mg dacomitinib with or without 30 mg paroxetine	Ph1 completed	NCT01318031
**Food-drug**	**AG-013736** **(axitinib)**	VEGFR, PDGFR, c-KIT inhibitor	Pharmacokinetic. To assess the food effect on drug levels.	Single doses axitinib in different fasting conditions	Ph1 completed	NCT00918632
**Cardiovascular Safety**	**SOM230** **(pasireotide)**	Somatostatin analogue	Safety. To assess cardiac repolarization.	Therapeutic pasireotide dose 600ug vs MTD vs placebo vs moxifloxacin	Ph1 completed	NCT01128192
**Bioavailability**	**SR13668**	AKT inhibitor	Pharmacokinetic. To determine the best bioavailable formulation.	Single doses of SR13668 testing different formulations	Ph1 completed	NCT00896207
**Bioequivalence**	**PF-05280014**	Anti-EGFR2 inhibitor	Pharmacokinetic. To assess bioequivalence between PF-05280014 and approved TTZ.	PF-05280014 vs TTZ-EU vs TTZ-US	Ph1 completed	NCT01603264
**Ph1 FIH**	**ARRY-142866** **(selumetinib)**	MEK1/2 inhibitor	Pharmacokinetic, safety and tolerability.	Single doses of 25 mg selumetinib, and with or without itraconazol or fluconazol	Ph1 completed	NCT02093728

*Abbreviations*: MTA (Molecularly Targeted Agents); HER (Human Epidermal growth factor Receptor); VEGFR (Vascular Endothelial Growth Factor Receptor); PDGFR (Platelet Derived Growth Factor Receptor); MTD (Maximum Tolerated Dose); EGFR2 (Epidermal Growth Factor Receptor 2); TTZ (TrasTuZumab); vs (versus); EU (European Union); US (United States).

## CONCLUSIONS

**Figure 1:**
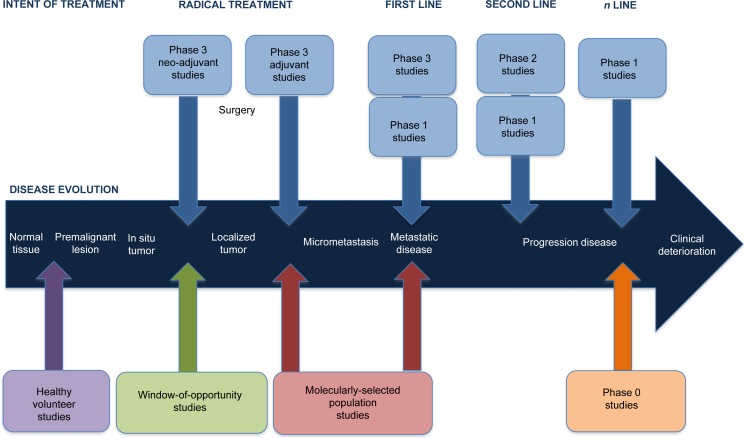
Depicting the journey of a cancer patient through the different new clinical trial designs in the Early Drug Development field The development of novel Molecularly Targeted Agents (MTA) has lead to the possibility of receiving these anti-cancer drugs in new patient populations. Noteworthy, one single patient could aim to participate in each one of the four types of studies here depicted throughout the evolution of his/her cancer. Being in good health status, the individual could participate in a Healthy Volunteer (HV) study, sometimes even decades before developing a premalignant lesion. Once diagnosed with an early invasive localized cancer, the patient may be considered candidate for receiving neo-adjuvant therapy according to the gold standard approach. It is in this same neo-adjuvant setting where the patient could consider to participate in a Window-Of-Opportunity (WOO) study, before proceeding with a radical surgery and adjuvancy. Whenever a metastatic disease recurrence occurs, the evaluation of a potential novel cancer biomarker for the tumor may significantly change the standard-of-care. If a predictive biomarker was identified, this patient may be prioritized for participating in a Molecularly Selected Population (MSP) trial, aiming to match the specific molecular aberration with a highly selective MTA. Should this patient progress despite receiving all the standard options, the patient may still be suitable for participating in a Phase 0 (Ph0) study for altruism reasons, even if no efficacy was pursuit.

The advent of new MTA has broadened the EDD landscape, not only in terms of expanding the portfolio of drugs, but also in changing the candidates exposed to these experimental therapies. It is clear that many different patient populations other than advanced cancer patients are currently exposed to MTA in their early development. Figure 1. Proposes a potential journey of a cancer patient through the conventional available therapies and their sequence, in parallel to the different new clinical trial designs currently available in EDD.

The appearance of these MTA has opened a field of possibilities towards a second paradigm in EDD, facing the physicians with the challenge of reconsidering “the old paradigm” that only heavily pre-treated cancer patients should be included in a FIH study. As MTA have reshaped clinical trials, there is a rising need to revisit the I/E criteria, considering that some of these criteria may be disproportionately restrictive and should be tailored to the potential risk/benefit for incurable cancer patients. Other key points would be how to homogenize the molecular pre-selection criteria for participating into MSP studies and the need of shortening the time of biomarker analysis, how to obtain higher purity tumor samples and how to overcome the intra-tumor heterogeinity. A coordination effort among multiple academic centers seems mandatory to guarantee a suitable patient identification and adequate accrual, especially considering these small patient populations. WOO studies in the neo-adjuvant setting will need to be considered as one of the most suitable arenas for obtaining more robust biomarker information. However, increasing the recruitment in WOO, Ph0 and HV studies, with no therapeutic intent at all, will certainly suppose a challenge for the Oncologists, as they will need to provide a correct comprehension of trial design and its objectives to the patients, ensuring they understand the risks whilst dealing with patient expectations. Physicians should keep in mind that Ph1 trials still need to focus on finding the appropriate dose that can be administered safely, with optimal efficacy and minimal side effects. Even if most Ph1 focus on early efficacy or biomarkers data nowadays, these should not shadow the primary objective of delineating the safety profile of new MTA, key for further development.

In conclusion, the particular toxicity profile of MTA has broaden the therapeutic window of the anti-cancer agents, although there is still a long way to go, and special caution will need to be taken when new therapeutic options are tested in these new settings. All these studies may represent a promising, fast and cost-effective method for developing new compounds that can overcome the traditional difficult processes for drug approval based on expensive larger randomized trials. Oncology is one of the fields with higher rates of failures at exceptionally high economic costs, a fact that mandates a profound review of the appropriate clinical trial design for regulatory approval of new MTA. MSP, WOO, Ph0 and HV studies may represent an optimal scenario for further progressing in the development of new drugs, in a timely-effective manner and reducing the economic burden for EDD field.
